# Genetic Variants Detected Using Cell-Free DNA from Blood and Tumor Samples in Patients with Inflammatory Breast Cancer

**DOI:** 10.3390/ijms21041290

**Published:** 2020-02-14

**Authors:** Jennifer S. Winn, Zachary Hasse, Michael Slifker, Jianming Pei, Sebastian M. Arisi-Fernandez, Jacqueline N. Talarchek, Elias Obeid, Donald A. Baldwin, Yulan Gong, Eric Ross, Massimo Cristofanilli, R. Katherine Alpaugh, Sandra V. Fernandez

**Affiliations:** Fox Chase Cancer Center, Philadelphia, PA 19111, USA; JenniferS.Winn@fccc.edu (J.S.W.); Zachary.Hasse@fccc.edu (Z.H.); Michael.Slifker@fccc.edu (M.S.); Jianming.Pei@fccc.edu (J.P.); sebasmfa@gmail.com (S.M.A.-F.); Jacqueline.Talarchek@fccc.edu (J.N.T.); Elias.Obeid@fccc.edu (E.O.); Donald.Baldwin@fccc.edu (D.A.B.); Yulan.Gong@fccc.edu (Y.G.); Eric.Ross@fccc.edu (E.R.); Massimo.Cristofanilli@nm.org (M.C.); R.Alpaugh@fccc.edu (R.K.A.)

**Keywords:** inflammatory breast cancer (IBC), cell-free DNA (cfDNA), next generation sequencing (NGS)

## Abstract

We studied genomic alterations in 19 inflammatory breast cancer (IBC) patients with advanced disease using samples of tissue and paired blood serum or plasma (cell-free DNA, cfDNA) by targeted next generation sequencing (NGS). At diagnosis, the disease was triple negative (TN) in eleven patients (57.8%), ER+ Her2- IBC in six patients (31.6%), ER+ Her2+ IBC in one patient (5.3%), and ER- Her2+ IBC in one other patient (5.3%). Pathogenic or likely pathogenic variants were frequently detected in *TP53* (47.3%), *PMS2* (26.3%), *MRE11* (26.3%), *RB1* (10.5%), *BRCA1* (10.5%), *PTEN* (10.5%) and *AR* (10.5%); other affected genes included *PMS1*, *KMT2C*, *BRCA2*, *PALB2*, *MUTYH*, *MEN1*, *MSH2*, *CHEK2*, *NCOR1*, *PIK3CA*, *ESR1* and *MAP2K4.* In 15 of the 19 patients in which tissue and paired blood were collected at the same time point, 80% of the variants detected in tissue were also detected in the paired cfDNA. Higher concordance between tissue and cfDNA was found for variants with higher allele fraction in tissue (AF_tissue_ ≥ 5%). Furthermore, 86% of the variants detected in cfDNA were also detected in paired tissue. Our study suggests that the genetic profile measured in blood cfDNA is complementary to that of tumor tissue in IBC patients.

## 1. Introduction

Inflammatory breast cancer is a very aggressive type of breast cancer with a poor prognosis. In the United States, it accounts for 2–6% of all patients with breast cancer [1,2,3]. The principal clinical symptoms of inflammatory breast cancer (IBC) are breast erythema, edema, peau d’orange, and dermal lymphatic invasion [4]. Despite its name, IBC is not associated with a profuse inflammatory response; the characteristic redness and swelling of the breast are due to obstruction of lymphatic channels in the dermis by tumor cells [5,6]. Although IBC is a rare clinical subtype of locally advanced breast cancer, it is responsible for approximately 10% of breast cancer-associated deaths annually in the US, which translates into 4000 deaths per year [2,6]. IBC is either stage III or IV at the time of diagnosis and the majority of patients have lymph node metastases, with one third of the patients having metastases in distant organs such as the brain, bone, other visceral organs, and soft tissue [6]. The median overall survival (OS) for patients with stage III IBC is 4.75 years, compared to 13.40 years in those with non-IBC. In stage IV disease, the median OS is 2.27 years in IBC patients versus 3.40 years in non-IBC patients [7,8]. Although IBC, like non-IBC breast cancers, is a heterogeneous disease and can occur as any of the four molecular subtypes, it is most commonly either human epidermal growth factor receptor 2 (HER2) overexpressive or triple negative (TN) [9,10]. TN breast cancer, which is defined by the absence of estrogen and progesterone receptors, and a lack of HER2 overexpression, has a poorer prognosis than other subtypes [11].

The principal objective of precision oncology is to improve the diagnosis and treatment of cancer patients, and its focus is increasingly turning to liquid biopsies such as cell-free DNA from blood. Due to tumor heterogeneity, the analysis of an individual tissue biopsy may not accurately reflect the genomic profile of a patient’s cancer and this could introduce bias to the selection and efficacy of personalized therapies. Instead, as DNA is released from multiple tumor regions into the bloodstream, cfDNA may reflect the aggregate genetic composition of intra-tumor heterogeneity [12,13] as well as metastases [14,15,16,17]. Furthermore, blood samples can be collected at multiple time points, facilitating longitudinal disease monitoring [18]. As IBC progresses rapidly, the use of blood cfDNA could be important in following disease progression and selection of new treatments. In the present work, we studied genetic variants in IBC patients using tissue and paired blood cfDNA samples to evaluate the concordance of observed mutation profiles. Genetic variants of clinical relevance were studied in TN and non-TN IBC.

## 2. Results

### 2.1. Patients and Samples

This retrospective study included 19 patients with IBC at advanced clinical stage (III or IV). All the patients were female, 15 were Caucasian (1 with Ashkenazi Jewish and 1 with Jewish heritages), 2 African-American, 1 Hispanic, and 1 Asian. The median age at IBC diagnosis was 47.6 years (range 32–69) with 3 patients > 60 years old. At the time of diagnosis, the disease was classified as ER- PR- Her2- (TN) in eleven patients (57.8%), ER+ Her2- in six patients (31.6%), ER+ Her2+ in one patient (5.3%), and ER- Her2+ in one other patient (5.3%). Changes in ER or Her2 receptor expression were seen in two patients: cancer cells from patient 14 of the ER+ Her2- group transformed into the TN phenotype at month 8, and patient 15 who had ER+ Her2+ disease at the time of diagnosis lost Her2 expression on a tumor biopsy performed in month 49 (Supplementary Data and Table S1). Four patients (#1, #9, #10, and #13) were initially diagnosed and treated for non-IBC breast cancer in the ipsilateral breast before being diagnosed with IBC (Supplementary Data). The median overall survival was 31 months for patients with TN IBC and 53 months for patients with non-TN IBC (Figure S1). IBC patients developed metastasis to the lung, bone, liver, and/or abdomen, and three patients (#1, #2, and #5) with TN disease developed brain metastases, one of whom (patient 5) also had leptomeningeal disease (Supplementary Data). Patient treatments and disease progressions are indicated for each patient (Supplementary Data).

Genomic variants were studied by next generation sequencing (NGS) in malignant tissue or cells from pleural effusions and paired cfDNA from peripheral blood (plasma/serum) in these 19 patients. At the time of sample collection, 17 patients had stage IV and 2 patients had stage III disease. A total of 40 samples were studied: skin breast biopsies from 9 patients, breast tissue biopsies from 3 patients, a lymph node from 1 patient, cells from malignant pleural effusions from 6 patients, and 21 samples of blood cfDNA. A panel of 93 breast cancer genes (Table S2) was used for targeted NGS; the data were analyzed using the Qiagen GeneGlobe portal. All variants detected in the 40 samples from the 19 IBC patients and ctDNA controls are shown in Table S3. A total of 379 variants were detected excluding 37 false positives variants (Table S3). False positives were considered variants with allele fraction (AF) > 0 in at least 18 of the 45 samples, including controls, which show low average AF across samples (< 0.2) and standard deviation < 0.04.

### 2.2. Clinically Relevant Variants in IBC

The QIAGEN Clinical Insight Interpreter (QCI) (Qiagen, Redwood City, CA, USA) was used to classify the clinical relevance of coding variants in the 19 IBC patients included in the study. Pathogenic, likely pathogenic, and variants of uncertain significance detected in malignant tissue/cells and blood cfDNA of patients with triple negative (Table 1) and non-TN (Table 2) IBC are shown. All the patients had received therapeutic treatment(s) at the time that samples were collected, except patient 5 and patient 19 at month 8 (both with TN disease), and patient 12 (ER+ Her2-) (Supplementary Data). For patient 1, cfDNA samples from month 12 (cfDNA1) and month 22 (cfDNA2) were studied and *PALB2* and *BARD1* variants were detected in both cfDNA, but the *RB1* mutation was only detected in blood cfDNA from month 22 (Table 1). The *RB1* splicing mutation was not present in the lymph node biopsy or blood cfDNA at month 12; this patient developed metastases in bone, liver, and brain that were detected by imaging at months 20–22 (Supplementary Data). Variants were also classified as biomarkers for a clinically available intervention (Tiers 1–3) (Table 1; Table 2) [19]. *BRCA1* and *BRCA2* pathogenic variants in patient 4 and patient 9 (Table 1) were classified as T1A since there are two poly (ADP-ribose) polymerase (PARP) inhibitors that have been approved by the FDA for patients with metastatic breast cancer who carry germline mutations in these genes [20,21].

**Figure 1:**
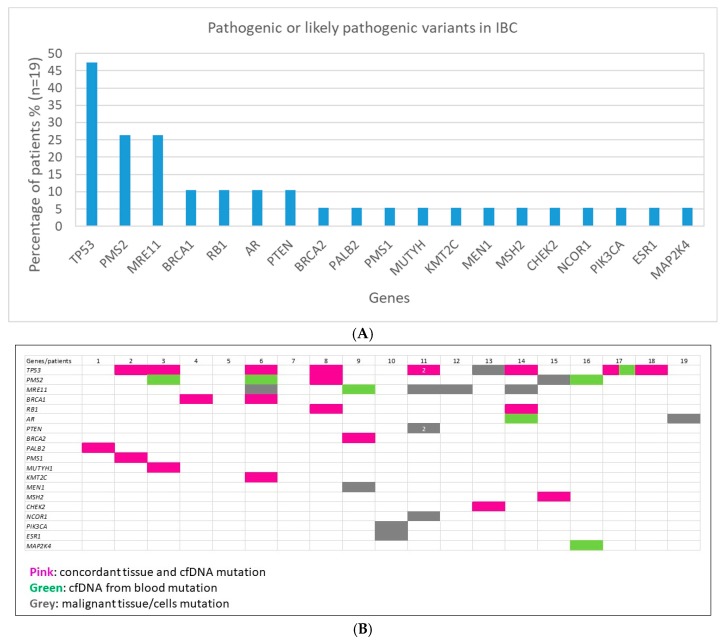
Genes with pathogenic or likely pathogenic variants in IBC. Genes with pathogenic or likely pathogenic variants in inflammatory breast cancer (IBC) patients (*n* = 19) are shown. (**A**) Percentage of IBC patients with pathogenic or likely pathogenic variants in the genes indicated. (**B**) Pathogenic or likely pathogenic variants detected in both tissue and cfDNA (in pink), only in cfDNA (in green) or only in malignant tissue/cells from pleural effusions are shown across all patients. In patient 11, two pathogenic *TP53* variants were detected in both tissue and cfDNA and two variants in *PTEN* (one pathogenic and another likely pathogenic) were detected in malignant cells from a pleural effusion. In patient 17, two *TP53* variants were detected: one pathogenic *TP53* variant in both tissue and cfDNA, and another *TP53* pathogenic variant detected only in cfDNA from plasma.

In the 19 IBC patients included in this study, pathogenic or likely pathogenic variants were most frequently detected in *TP53* (9/19 patients; 47.3%), *PMS2* (5/19; 26.3%), *MRE11* (5/19; 26.3%), *BRCA1* (2/19; 10.5%), *RB1* (2/19; 10.5%), *AR* (2/19; 10.5%), and *PTEN* (2/19; 10.5%) in malignant tissue/cells and/or cfDNA samples; others were detected in *BRCA2*, *PALB2*, *PMS1*, *MUTYH*, *KMT2C*, *MEN1*, *MSH2*, *CHEK2*, *NCOR1*, *PIK3CA*, *ESR1* and *MAP2K4* (Figure 1A). From 41 pathogenic or likely pathogenic variants detected in 19 IBC patients, 21 (51.2%) were detected in both malignant tissue/cells and paired cfDNA (Figure 1B); 13 (31.7%) were detected only in tissue, and 7 (17.1%) were only detected in cfDNA (Figure 1B). Four pathogenic variants were only detected in tissue although they were at high AF (> 10%); these pathogenic variants were in *MEN1* in patient 9 (Table 1); *PIK3CA* and *ESR1* in patient 10 (Table 2); and *NCOR1* in patient 11 (Table 2).

Variants with AF ~50% in both tissue and matching cfDNA from blood were detected in: *BRCA1* (patient 4; Figure 1B and Table 1), *BRCA2* (patient 9; Figure 1B and Table 1) and *TP53* (patient 14; Figure 1B and Table 2), all of which were putative germline variants (AF ~ 50% in cfDNA and paired tissue). In this retrospective study, leukocytes, fibroblasts, or normal tissue samples were not available to identify germline variants. However, in patients 4 and 9, those *BRCA* variants were confirmed to be of germline origin by clinical genetic tests performed at the time of diagnosis (Supplementary Data).

### 2.3. Concordance of Variants in Tissue and Paired Blood cfDNA

**Figure 2:**
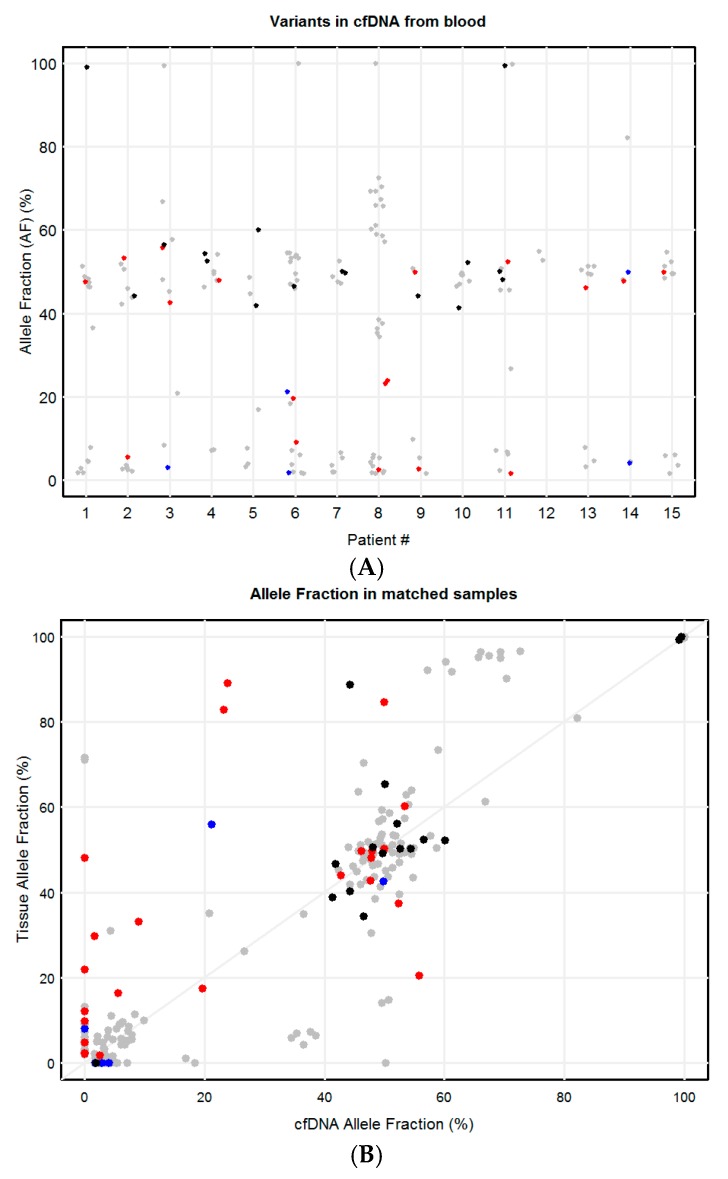
Allele fraction (AF) of genetic variants detected in blood cfDNA of 15 IBC patients with paired tissue samples in which blood and tissue were collected at the same time-point. Variants are shown for patients 1 to 15. (**A**) AF of variants detected using cfDNA from blood; (**B**) AF of variants detected in malignant tissue/cells and paired cfDNA from blood. In red, pathogenic variants; in blue, likely pathogenic variants; in black, variants of uncertain significance; in grey, benign or likely benign variants.

To study concordance between variants in tissue and paired blood cfDNA, only those patients in which tissue and blood were collected at the same time point were considered. From the 19 patients studied, tissue and blood samples were collected at the same time-point in 15 patients (#1 to #15). cfDNA profiles revealed three groups: (1) a low allele fraction (AF) group of variants; (2) a middle group of variants centered around 50% AF; and (3) a high group of variants centered around 100% AF, likely representing homozygous germline variants (Figure 2A). Some variants were detected in both tumor tissue and paired blood cfDNA, while others were detected in the tumor biopsy but not in the cfDNA; still others were detected in cfDNA from blood but not in the tissue biopsies or cells from malignant pleural fluids (Figure 2B).

**Figure 3:**
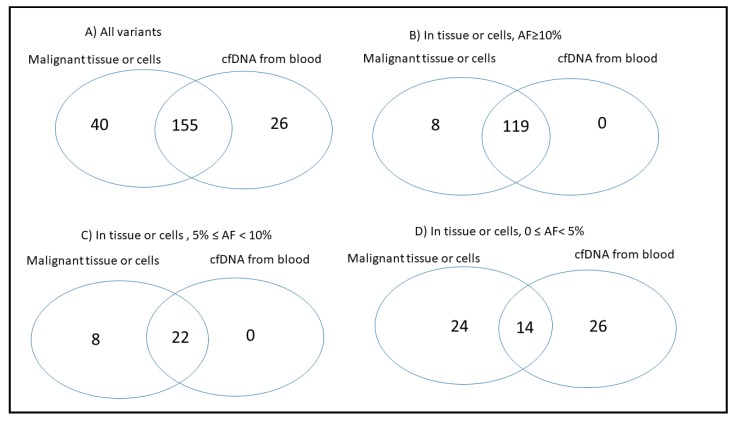
Variant concordance between malignant tissue/cells and paired cfDNA from blood (plasma or serum) in 15 patients in which blood and tissue samples were collected at the same time-point. (**A**) Considering all the variants; (**B**) For variants with AF ≥ 10% in malignant tissue or cells; (**C**) For variants with 5% ≤ AF < 10% in malignant tissue or cells; (**D**) For variants with AF < 5% in malignant tissue or cells.

To study concordance of variants between tissue and paired cfDNA, data from these 15 patients were extracted from Table S3, and germline variants observed in the overall population with an allele frequency of 2.5% or greater in the Genome Aggregation Database (gnomAD) [22] were excluded from the data (Table S4). We considered concordant alterations only when the exact same sequencing alteration was present in both malignant tissue/cells and paired cfDNA from blood. Variants causing coding changes of uncertain significance, and those pathogenic, likely pathogenic, benign, and likely benign were considered in studying concordance between tissue and paired cfDNA samples; synonymous DNA alterations were excluded. The number of variants detected in both tumor tissue and paired cfDNA (concordant variants), or observed only in the tumor biopsy or only in cfDNA were counted for each patient (Table S5). A total of 221 variants were identified in the 15 patients from any source (Table S5). Of the 195 variants detected in malignant tissue/cells, 155 were also observed in the cfDNA (sensitivity = 79.5%) (Table S5 and Figure 3A). From 181 variants detected in blood cfDNA, 155 variants were also detected in the tissue (Table S5 and Figure 3A); the probability that a variant detected in the blood was also seen in malignant tissue/cells (positive predictive value, PPV) was 85.6% (Figure 3A). A total of 26 variants (14.4%) detected in blood cfDNA were not present in the paired malignant tissue or cells from pleural effusions (Figure 3A).

For variants with AF ≥ 10% in malignant tissue or cells, 93.7% were also detected in paired cfDNA (119 of 127, Figure 3B). For variants with 5% ≤ AF ˂ 10% in malignant tissue or cells, 73.3% were also detected in paired cfDNA (22 of 30, Figure 3C). However, for variants with tissue AF < 5%, only 36.8% were also detected in paired cfDNA (14 of 38, Figure 3D).

## 3. Discussion

Genetic studies using cfDNA from blood in IBC patients may allow the profiling of genetic changes over time, enabling the use of more efficient therapies in this rapidly progressing disease. cfDNA from blood has been evaluated to determine whether it can be used as an alternative to tissue biopsies in several types of cancers. Our studies showed a high concordance between genetic variants detected in tumor tissue and blood cfDNA from IBC patients with advanced disease, in particular for those variants with AF > 5% in tissue. For variants with AF ≥ 10% in malignant tissue or cells, 93.7% were also detected in matching blood cfDNA, and for variants with 5% ≤ AF < 10% in malignant tissue or cells, 73.3% were also detected in paired cfDNA from blood. However, for variants with AF < 5% in tissue or cells, only 36.8% were also detected in cfDNA. Furthermore, 85.6% of the variants detected in blood cfDNA were also detected in paired malignant tissue/cells. Previous studies in other types of cancer have shown a concordance of 50–88% when comparing tumor DNA and blood cfDNA using NGS [23,24]. Low concordance (10.8–15.1%) was found in an NGS study that included 45 breast cancer patients, 34 of whom had IBC [25]. The low concordance could be explained by the fact that two different platforms, with different sequencing techniques, were used to study tissue and paired cfDNA [25]. In that study, the FoundationOne (Foundation Medicine) test was used for formalin-fixed, paraffin-embedded specimen, and cfDNA was tested using the Guardant360 (Guardant Health) platform; concordance was particularly low for copy number variants (CNVs) [25]. The numbers of genes tested in the FoundationOne and Guardant 360 panels were 315 and 70, respectively. Kuderer et al. found also low concordance between tumor and paired blood cfDNA using these two platforms [26], however, in both studies, the concordance increased after restricting comparisons to variants found in the cfDNA at AF greater than 1% [25,26]. In our work, we used the same platform to study tissue and blood cfDNA samples, and only variants with AF ≥ 1.5% in cfDNA were considered. We studied single nucleotide variants (SNVs) and small indels (insertions and deletions), but did not include CNVs in the analysis. Although combining liquid biopsy with NGS technology provides a noninvasive method to analyze numerous cancer-related genes in a single assay, detecting low AF of SNVs through NGS still presents significant challenges due to the high rates of false positives when tumor DNA is in low concentration as in the case of cfDNA.

From the genetic variants detected in blood, 14.3% of them were not present in paired tissue samples. Metastatic lesions have a genomic fingerprint that may evolve and become discordant from the primary tumor [27]. Most of the patients in this study had stage IV disease and most of them developed distal metastases to the lung, bone, liver, and/or abdomen that could explain the presence of variants in blood cfDNA coming from these metastatic sites. cfDNA may be derived from a primary tumor, metastatic lesions, or circulating tumor cells (CTCs) [28]; both apoptosis and necrosis, alongside active secretion, play important roles in the cfDNA presence in liquid biopsies [29,30].

In addition to cfDNA found in plasma and serum, cfDNA in urine has shown promise as a biomarker for certain cancers. For example, in patients with non-muscle-invasive bladder cancer, high levels of cfDNA were found in urine samples in patients with progressive disease, including samples from patients where levels of cfDNA were low in plasma [31]. Moreover, in a genomic analysis of urine cfDNA in patients with urothelial bladder cancer, there was a high rate of concordance between mutations found in urine cfDNA and tumor tissue [32]. Saliva cfDNA was used to study variants in patients with oral cancer [33]. In patients with brain tumors, ctDNA in blood is rarely found, presumably due to the blood–brain barrier [34]. Cerebrospinal fluid (CSF) ctDNA was identified in primary and metastatic brain tumors [17]. Three patients from our cohort study developed brain metastases, one of whom also had leptomeningeal disease. Genomic profiling of CSF might guide clinical decisions in IBC patients who develop brain or leptomeningeal metastasis [35].

In the present work, pathogenic or likely pathogenic variants were most frequently detected in *TP53* (47.3%), *PMS2* (26.3%), *MRE11* (26.3%), *BRCA1* (10.5%), *RB1* (10.5%), *AR* (10.5%) and *PTEN* (10.5%); others in *PMS1*, *KMT2C*, *BRCA2*, *PALB2*, *MUTYH*, *MEN1*, *MSH2*, *CHEK2*, *NCOR1*, *PIK3CA*, *ESR1* and *MAP2K4* were detected in 5.3% of patients. Most of these variants correspond to proteins involved in DNA repair (PMS2, MRE11, BRCA1, BRCA2, PALB2, PMS1, MUTYH, CHEK2, MSH2) and control of the cell cycle (TP53, RB1, CHEK2). Deficient DNA repair and control of cell cycle would contribute to disease progression. In a recent study of 101 untreated primary IBC tumors aggregated from four public datasets, Bertucci et al. showed that the genomic profile of IBC is different from non-IBC breast cancer [36]. Genes involved in DNA repair were found more frequently altered in IBC than in non-IBC breast cancer [36]. *TP53* was found to be the most frequently altered gene in IBC and its rate of mutation in IBC was found to be significantly higher than in non-IBC patients [36,37,38]. Matsuda et al. found that *TP53* was altered in 75% of IBC (18/24 patients) and in 28.2% (106/376 patients) of non-IBC patients [38]. Liang et al. found alterations in *TP53* in 43% (67/156) of the IBC patients (61/197) and 31% of the non-IBC breast cancer patients [37]. The likely pathogenic variant androgen receptor (AR) c.170T > A (p. L57Q) detected in two IBC patients from our study represents a missense mutation in the amino-terminal domain of the AR with partial loss of function of the protein. The AR, like the estrogen receptor (ER) and the progesterone receptor (PR), is a member of the steroid hormone receptor family. There is a significant association between AR and ER expression in breast carcinoma [39]. In general, AR-positive status is significantly associated with better clinical outcomes than AR-negative tumors; however, in some studies, the significant prognostic relevance of AR was observed in ER-positive tumors, but not in ER-negative tumors or triple negative tumors [39]. In another study, AR negativity was associated with a greater frequency of recurrence and distant metastasis in triple negative tumors [40]. IBC patients who were found to have AR-negative/ER-negative tumors had the worst survival outcomes compared to patients who had tumors that exhibited other AR/ER combinations [41].

Importantly, although our studies showed high concordance between variants detected in tissue and paired cfDNA from blood, some pathogenic variants detected at high AF in tissue were not detected in cfDNA. These variants were found in *MEN1*, *PIK3CA* and *ESR1*, suggesting that the information detected from blood cfDNA could, at most, be complementary to the variants detected in tissue.

Although the number of samples studied in the present work was low, it must be taken into consideration that IBC is a rare disease that accounts for only 2–5% of all patients with breast cancer. Many inflammatory breast cancer genetic studies face challenges of a paucity of samples given the rarity of the disease. Five NGS-based studies have been published regarding IBC using tissue samples in which the number of genes tested varied between 50 to 255 [37,38,42,43,44]. In these studies, targeted NGS [37,38,43,44] and whole-exome sequencing [42] were used.

The fact that IBC patients tend to be younger than other breast cancer patients (52 years in IBC vs. 57 in non-IBC) has suggested a genetic component in the etiology of IBC [45]. In a recent study of 368 IBC patients, it was found that 14.4% carried pathogenic germline variants [46]. *BRCA1* and *BRCA2* germline pathogenic variants were found in 7.3% of the IBC patients, 6.3% had a mutation in other cancer genes (*PALB2*, *CHEK2*, *ATM* and *BARD1*), and 1.6% had a germline pathogenic variant in other genes not related with breast cancer [46]. In this study, putative pathogenic variants with AF~50% in both tissue and paired cfDNA were detected in *BRCA*, *BRCA2* and *TP53* in three patients; those *BRCA1* and *BRCA2* variants were confirmed to be of germline origin since those patients had clinical genetic tests performed. Pathogenic germline variants in *BRCA1* and *BRCA2* genes are highly penetrant, conferring a risk that is more than four times that of the non-mutated population [47]. The patient who carried the *TP53* mutation at high AF had also Ehlers–Danlos syndrome and a first degree relative with prostate cancer. A putative germline variant of uncertain significance found commonly in patients from this study was *BARD1* V507M, which was carried by 7 of the 19 patients. BARD1 (BRCA1-associated ring domain) encodes a protein which interacts with the N-terminal region of BRCA1. BARD1 is vital in the rapid relocation of BRCA1 to DNA damage sites [48] and has been associated with increased risk of breast cancer in postmenopausal Japanese women [49]. Although this alteration has not been associated with familial or sporadic breast cancer in other populations [50], a statistically significant association of this variant with high-risk neuroblastoma has been demonstrated [51,52]. One limitation of our study design is that it was retrospective and control samples for germline profiling such as leukocytes, fibroblasts, or other normal tissue samples were not available. Internal validation in future studies will provide a more accurate estimate of the expected germline mutation prevalence in IBC, and somatic mutations that could arise from clonal hematopoiesis that could confound cfDNA analysis [53,54].

Our results suggest that the information regarding genetic variants in blood cfDNA from IBC patients is complementary to the variants detected using malignant tissue samples. Further studies are ongoing to improve the sensitivity of these assays, such as deeper sequencing using a different panel to increase the sensitivity of the assays in cfDNA from blood. Prospective studies are necessary in order to distinguish germline and somatic variants in IBC.

## 4. Materials and Methods

### 4.1. Patient Cohort

The samples used in this study were collected from 19 IBC patients who were treated at Fox Chase Cancer Center (FCCC) between 2010–2012. This study was approved by both the research review committee (RRC) and the institutional review board at FCCC (IRB 10-826 approved on 24/08/2010). Patients signed an informed consent and HIPAA certification from the Human Subject Protection Committee prior to sample collection. Retrospective chart reviews were performed to assess age at diagnosis, hormone receptor subtype, treatments, disease progression, and family history of cancers (Supplementary Data and Table S1). Overall survival (OS) was calculated from the day of diagnosis to the day of last follow-up or death.

### 4.2. Sample Collection

Cells from malignant pleural effusions were centrifuged at 1000× *g* for 10 min to create a cell pellet. The pellets were then suspended in 0.2% NaCl and an equal volume of 1.6% NaCl was added to induce red blood cell (RBC) hemolysis. This mixture was centrifuged again at 1000× *g* for 10 min, and the hemolysis step was repeated to remove all the remaining RBCs. Finally, the cell pellets were washed with phosphate-buffered saline and the cell pellets were preserved in optimal cutting temperature compound (OCT) at −80 °C. Some tumor tissue biopsies were preserved in OCT at −80 °C, while formalin fixed paraffin embedded (FFPE) blocks were prepared for others. For serum samples, blood was allowed to clot for 30–60 min and centrifuged at 2000× *g* for 20 min, after which sera was then separated. To isolate plasma, blood was placed into ethylene diamine tetra-acetic acid (EDTA) tubes and centrifuged for 15 min at 2000× *g*, and plasma was then separated. Blood samples were processed within an hour from the time of collection.

### 4.3. DNA Isolation

For the plasma and serum samples, the maximum volume available (3–6 mL) was used to isolate cell free DNA (cfDNA). Samples were centrifuged at 16,000× *g* for 10 min and the QIAamp MinElute ccfDNA Midi kit (Qiagen, Redwood City, CA, USA) was used for cfDNA isolation. Genomic DNA was isolated from either the OCT-preserved or from formalin fixed paraffin embedded (FFPE) blocks from tumor cells and tissues. Two 10 µm unstained sections were cut from FFPE blocks and used for DNA isolation. Genomic DNA from the frozen samples was isolated using the QIAamp DNA micro kit (Qiagen, Redwood City, CA, USA), and the QIAamp DNA FFPE tissue kits (Qiagen, Redwood City, CA, USA) were used for FFPE sections. The isolated DNA samples were quantified using a Qubit fluorometer (Thermos Fisher, Waltham, Massachusetts, USA) and then used to prepare libraries for targeted next generation sequencing.

### 4.4. Library Preparation

Libraries were prepared using the QIAseq Targeted DNA Panel, Human Breast Cancer Panel (DHS-001Z, Qiagen), and the QIAseq 12-index (48) (Qiagen, Redwood City, CA, USA). The DHS-001Z breast cancer panel covers 93 breast cancer relevant genes (Table S2) and contains 4831 primers. It is able to detect both SNVs and small indels; copy number variants were not studied. A total of 10–100 ng of cfDNA from plasma or serum (the maximum available; Table S6), 50 ng of DNA from tissue biopsies or cells from OCT-preserved malignant effusions, and 40–250 ng of DNA from FFPE tissue were used for NGS-library preparation. As control, 50 ng of circulating tumor DNA (ctDNA) reference material v2 of AF 2% (Cat# 0710-0203, SeraCare, Milford, MA, USA) was used to prepare control libraries. Wild-type Seraseq ctDNA Reference Material v2 WT (Cat # 0710-0208, SeraCare, Milford, MA, USA) was used to prepare the allele fraction dilutions of 1%, 0.5%, and 0.25% from the 2% ctDNA reference material. These ctDNA controls were run in order to establish the ability to detect the reference variants and to determine the lower limit of detection of our assays.

### 4.5. Next Generation Sequencing and Data Analysis

Library preparation included sequence barcodes to discriminate samples and unique molecular indices to identify polymerase chain reaction (PCR) duplication. Four libraries were pooled together per flow cell and sequenced using Illumina MiniSeq with high-output kits (Illumina, San Diego, CA, US) producing an average of 5,389,641 total reads per sample and 781 paired-end reads per targeted region (Table S6). The data were analyzed using Qiagen GeneGlobe bioinformatics tools. Variants were filtered out if the within-sample allele frequency was less than 2% for tissue samples and less than 1.5% for blood cfDNA, or if fewer than ten reads were observed for the variant. Regions sequenced with fewer than 230 total reads were not considered for variant detection. Synonymous coding variants were filtered out and only exonic and/or splicing variants were retained (this excluded intronic events, but also events labeled intergenic, associated with 3′ or 5′ UTR, etc.) (Table S3). In Table S3, false positives were considered variants with AF > 0 in at least 18 of the 45 samples, including controls, which show low average AF across samples (< 0.2) and standard deviation < 0.04. Allele fraction for a specific variant can be defined as the as the number of times that variant is observed divided by the total number of reads of that region after sequencing. Allele fraction (AF) is distinct from allelic frequency, which describes the frequency of an allele in a population. Control samples such as leukocytes, fibroblasts, or normal tissue samples were not available to perform validation of germline variants.

### 4.6. Variant Classification

Qiagen Clinical Insight Interpret (QCI Interpret; Qiagen, Redwood City, CA, USA) was used to annotate variants. QCI Interpret evaluates variants by matching to a database of published supporting evidence and returns classifications using consensus guidelines for variants predicted to be pathogenic or likely pathogenic, benign or likely benign, or of uncertain significance [19,55]. It also classifies variants according to its clinical actionability; clinical actionability subcategories are provided based on levels of evidence according to the guidelines [19,55]. Tier 1 indicates strong clinical significance, with level A variants to predict response or resistance to therapies approved by the FDA for specific types of tumors (here breast cancer), and level B variants predicted to affect therapy based on well-powered studies or smaller studies that are confirmed or reproduced by different groups [19,55]. Tier 2 indicates potential clinical significance, and includes level C which indicates evidence of an effect on FDA-approved therapies for different tumor types or investigational therapies (2C: off-label treatments), while Tier 2D variants are supported by evidence from preclinical trials or a few case reports [19,55].

### 4.7. Concordance of Genetic Variants in Tissue and Paired cfDNA from Plasma/Serum

The time of collection of blood matched to the time of collection of the tissue in 15 patients: Patient 1 (T77549), Patient 2 (C65525), Patient 3 (L67523), Patient 4 (I74311), Patient 5 (K75070), Patient 6(B79071), Patient 7 (S80274), Patient 8 (K93878), Patient 9 (D89802), Patient 10 (M71182), Patient 11 (M85099), Patient 12 (P73793), Patient 13 (B68225), Patient 14 (B78899), and Patient 15 (B62630). To study concordance of variants between tissue and paired cfDNA from peripheral blood (plasma or serum), variants data from these 15 patients were extracted from Table S3. Germline variants observed in the overall population with an allele frequency of 2.5% or greater in the Genome Aggregation Database (gnomAD) [22] were excluded from the data. Once variants of interest were identified (Table S4), concordance between tissue and paired cfDNA was calculated. Concordance was defined as detecting an identical sequencing variant in tissue and paired cfDNA from blood; variants of uncertain significance, and those pathogenic, likely pathogenic, benign, and likely benign were used for concordance calculations and synonymous DNA alterations were excluded. Sensitivity and positive predictive value (PPV) were calculated.

### 4.8. Statistical Considerations

Standard descriptive statistics were used to characterize the study population with respect to demographic, clinical, pathologic, and biomarker data. The level of concordance between variants detected in malignant tissue and blood cfDNA was evaluated by computing (1) the proportion of variants observed in the malignant tissue/cells that were also detected in patient’s matched blood cfDNA sample, and (2) the proportion of variants detected in blood cfDNA that were also observed in the matched tissue sample. The proportion of variants identified in blood cfDNA that were not present in the paired malignant tissue or cells from pleural effusions was also estimated. The median overall survival (OS) time for patients with, and without triple negative IBC was estimated using the methods of Kaplan and Meier.

### 4.9. Data Availability

The datasets generated during and/or analyzed during the current study are available from the corresponding author on reasonable request.

## Figures and Tables

**Table 1:** Clinically relevant variants in triple negative IBC. Variants were classified as pathogenic (in red), likely pathogenic (in blue) or variants of uncertain significance (in black). Variants were also classified according to its actionability: T1A indicates strong clinical significance; T2C indicates potential clinical significance and FDA-approved therapies for different tumor types or investigational therapies. For each variant, its allele fraction (AF) is indicated in parenthesis. The samples used to study genomic variants, collection time from disease onset, and stage of the disease at the time of sample collection are indicated. (1) For patient 1, cfDNA samples from month 12 (cfDNA1) and month 22 (cfDNA2) were studied and *PALB2* and *BARD1* variants were detected in both, but the *RB1* mutation was only detected in cfDNA from month 22. All the patients were pre-treated except patient 5 (month 1) and patient 19 at month 8 that did not receive any treatment at those times. (**) In patients 4 and 9, the *BRCA* variants were confirmed to be of germline origin.

Patient ID	Tissue Biopsy	Cell-Free DNA from Blood
Patient 1 (T77549)	Lymph Node- Month 12 (Stage IV)	Plasma from Month 12 (cfDNA1) and Month 22 (cfDNA2)
	PALB2 c.1317delG p. G439fs*12 (43%) T2C BARD1 c.1518_1519delTGinsCA p. V507M (99%)	PALB2 c.1317delG p. G439fs*12 (51%) T2C BARD1 c.1518_1519delTGinsCA p. V507M (99%) RB1 splice 607+1 G>C (11%) (only in cfDNA month 22) (1)
Patient 2 (C65525)	Breast tissue biopsy - Month 33 (Stage IV)	Serum- Month 33
	PMS1 c.605G>A p. R202K (60%) BARD1 c.1518_1519delTGinsCA p. V507M (40%) TP53 c.721_722insA p. S241fs*23 (16%) T2C	PMS1 c.605G>A p. R202K (53%) BARD1 c.1518_1519delTGinsCA p. V507M (44%) TP53 c.721_722insA p.S241fs*23 (5.59%) T2C
Patient 3 (L67523)	Cells from malignant pleural effusion -Month 12 (Stage IV)	Serum - Month 12
	MUTYH c.1187G>A p. G396D (44%) SYNE1 c.23102G>A p. R7701Q (52%) TP53 c.375+1G>T (21%) T2C	MUTYH c.1187G>A p. G396D (43%) SYNE1 c.23102G>A p. R7701Q (56%) TP53 c.375+1G>T (56%) T2C PMS2 c.89A>C p. Q30P (2.97%) T2C
Patient 4 (I74311)	Skin breast biopsy- Month 17 (Stage IV)	Serum - Month 17
	** BRCA1 c.68_69delAG p. E23fs*17 (50%) T1A EXT2 c.520A>C p.M174L (50%) PTGFR c.465G>A p.M155I (50%)	** BRCA1 c.68_69delAG p. E23fs*17 (48%) T1A EXT2 c.520A>C p. M174L (54%) PTGFR c.465G>A p. M155I (53%)
Patient 5 (K75070)	Skin breast biopsy- Month 1 (Stage III)	Plasma - Month 1
	BARD1 c.1518_1519delTGinsCA p. V507M (52%) ATM c.5042T>C p. I1681T (47%)	BARD1 c.1518_1519delTGinsCA p. V507M (60%) ATM c.5042T>C p.I1681T (42%)
Patient 6 (B79071)	Skin breast biopsy- Month 35 (Stage IV)	Serum- Month 35
	SYNE1 c.14666C>G p. S4889C (34%) TP53 c.690_702delCACCATCCACTAC p. I232fs*11 (56%) T2C BRCA1 c.5278-1G>T (33%) T1A KMT2C c.2976+1G>A (17%) MRE11 c.1532delA p. N511fs*13 (2.23%) T2C	SYNE1 c.14666C>G p. S4889C (47%) TP53 c.690_702delCACCATCCACTAC p. I232fs*11 (21%) T2C BRCA1 c.5278-1G>T (9.05%) T1A KMT2C c.2976+1G>A (20%) PMS2 c.89A>C p. Q30P (1.81%) T2C
	Skin breast biopsy- Month 13 (Stage IV)	Serum- Month 13
Patient 7 (S80274)	BARD1 c.1518_1519delTGinsCA p. V507M (49%)	BARD1 c.1518_1519delTGinsCA p. V507M (50%) ESR1 c.805C>T p.R269C (50%)
Patient 8 (K93878)	Cells from malignant pleural effusion - Month 44 (Stage IV)	Serum- Month 44
	RB1 c.2336T>A p. L779* (89%) TP53 c.294_310 delTTCCCAGAAAACCTACC p. S99fs*44 (83%) T2C PMS2 c.1239delA p. D414fs*34 (1.8%) T2C	RB1 c.2336T>A p. L779* (24%) TP53 c.294_310 delTTCCCAGAAAACCTACC p. S99fs*44 (23%) T2C PMS2 c.1239delA p. D414fs*34 (2.53%) T2C
	Breast tissue biopsy- Month 3 (Stage IV)	Serum - Month 3
Patient 9 (D89802)	** BRCA2 c.7976G>A p. R2659K (50%) T1A BARD1 c.1518_1519 delTGinsCA p. V507M (89%) MEN1 c.1471G>T p. E491* (71%)	** BRCA2 c.7976G>A p. R2659K (50%) T1A BARD1 c.1518_1519 delTGinsCA p. V507M (44%) MRE11 c.1532delA p. N511fs*13 (2.64%) T2C
Patient 18 (E78569)	Cells malignant pleural effusion-Month 26 (Stage IV)	Plasma- Month 21 (Stage IV)
	KMT2C c.10432C>G p. Q3478E (43%) SYNE1 c.12149_12150delAGinsGT p. K4050S (53%) NBN c.1729G>T p. D577Y (78%) TP53 c.817C>T p. R273C (58%) T2C	KMT2C c.10432C>G p. Q3478E (50%) SYNE1 c.12149_12150delAGinsGT p. K4050S (51%) NBN c.1729G>T p. D577Y (58%) TP53 c.817C>T p. R273C (6.77%) T2C
Patient 19 (S73507)	Skin breast biopsy- Month 8 (Stage IV)	Plasma- Month 15 (Stage IV)
	BARD1 c.2300_2301delTG p. V767fs*4 (44%) AR c.170T>A p. L57Q (4.86%)	BARD1 c.2300_2301delTG p. V767fs*4 (41%)

**Table 2:** Clinically relevant variants in non-triple negative IBC. Variants are classified as pathogenic (in red), likely pathogenic (in blue) or variants of uncertain significance (in black). Variants are also classified according to its actionability: T2C indicates potential clinical significance and FDA-approved therapies for different tumor types or investigational therapies; T2D variant with potential clinical significance with evidence from preclinical trials or few cases reports without consensus. For each variant, its allele fraction (AF) is indicated in parenthesis. The samples used to study genomic variants, time of collection from disease onset and stage of the disease at the time of sample collection are indicated. (1) variants of clinical significance were not detected. (**) Indicated putative germline variant. All patients, except patient 12, were pre-treated at the time that the samples were collected for the study.

Patient ID	Tissue Biopsy	Cell-Free DNA from Blood
Patient 10 (M71182)	Skin breast biopsy- Month 54 (Stage IV)	Plasma - Month 54
	ErbB2 c.2689C>T p. R897W (56%) XRCC2 c.622_624delGAA p. E208del (39%) PIK3CA c.3140A>G p. H1047R (48%) T2C ESR1 c.1613A>G p. D538G (22%) T2D	ErbB2 c.2689C>T p. R897W (52%) XRCC2 c.622_624delGAA p. E208del (41%)
Patient 11 (M85099)	Cells from malignant pleural fluid- Month 29 (Stage IV)	Serum- Month 29
	BARD1 c.1518_1519delTGinsCA p. V507M (100%) CDKN2A c.442G>A p. A148T (65%) SYNE1 c.12149_12150delAGinsGT p. K4050S (51%) TP53 c.541C>T p. R181C (37%) T2C TP53 c.818G>A p. R273H (30%) T2C NCOR1 c.842+1G>A (12%) PTEN c.955_958delACTT p. T319* (9.83%) T2C MRE11 c.1532delA p. N511fs*13 (2.23%) T2C PTEN c.843_858delAGGACCAGAGGAAACC p.G282fs*4 (8.11%) T2C	BARD1 c.1518_1519delTGinsCA p. V507M (100%) CDKN2A c.442G>A p. A148T (50%) SYNE1 c.12149_12150delAGinsGT p. K4050S (48%) TP53 c.541C>T p. R181C (52%) T2C TP53 c.818G>A p. R273H (1.65%) T2C
Patient 12 (P73793)	Breast tissue biopsy- Month 1 (Stage IIIB)	Serum (cfDNA2) - Month 1
	MRE11 c.1532delA p.N511fs*13 (2.29%) T2C	(1)
Patient 13 (B68225)	Skin breast biopsy- Month 19 (Stage IV)	Serum- Month 19
	CHEK2 c.444+1G>A (50%) T2C TP53 c.286dupT p. S96fs*53 (4.88%) T2C	CHEK2 c.444+1G>A (46%) T2C
Patient 14 (B78899)	Skin breast biopsy - Month 8 (Stage IV)	Plasma- Month 8
	**TP53 c.796G>A p. G266R (48%) T2C RB1 c.131_132insTT p. V45fs*21 (43%) MRE11 c.1532delA p. N511fs*13 (2.11%) T2C	**TP53 c.796G>A p. G266R (48%) T2C RB1 c.131_132insTT p. V45fs*21 (50%) AR c.170T>A p. L57Q (4.06%)
Patient 15 (B62630)	Cells from malignant pleural effusion- Month 50 (Stage IV)	Serum - Month 50
	MSH2 c.435T>G p. I145M (85%) T2C PMS2 c.1239delA p. D414fs*34 (2.34%) T2C	MSH2 c.435T>G p. I145M (50%) T2C
Patient 16 (T73616)	Cells from malignant pleural fluid - Month 9 (Stage IV)	Plasma- Month 3 (Stage IV)
	SYNE1 c.12149_12150delAGinsGT p. K4050S (50%) MYC c.1085C>T p. S362F (50%)	SYNE1 c.12149_12150delAGinsGTp. K4050S (59%) MYC c.1085C>T p. S362F (52%) MAP2K4 c. 400C >T p. R134W (26%) PMS2 c.89A>C p.Q30P (1.92%) T2C
Patient 17 (S69308)	Skin breast biopsy- Month 31 (Stage IV)	Plasma -Month 25 (Stage IV)
	IRAK4 c.529A>G p. T177A (61%) TP53 c.602delT p. L201fs*46 (57%) T2C	IRAK4 c.529A>G p. T177A (57%) TP53 c.602delT p. L201fs*46 (16%) T2C TP53 c.638G>A p. R213Q (4.81%) T2C

## Supplementary Materials

Supplementary materials can be found at https://www.mdpi.com/1422-0067/21/4/1290/s1. Supplementary Data—Patient clinical history; Figure S1—Kaplan-Meier survival curves in IBC; Table S1—Patient demographics and survival; Table S2—Breast cancer panel DHS-001Z; Table S3—Genetic variants in all the samples of the 19 IBC patients; Table S4—Genetic variants in 15 patients with matched blood cfDNA and tissue samples collected at the same time point; Table S5—Number of variants in tissue and cfDNA in patients in which both samples were collected at the same time point; TableS 6—Quantity of cfDNA used to prepared libraries and total number of reads for each sample.

## Abbreviations

ACD	acid citrate dextrose
AF	allele fraction
AR	androgen receptor
BC	breast cancer
cfDNA	cell-free DNA
ctDNA	cell-tumor DNA
CH	clonal hematopoiesis
CI	confidence interval
CSF	cerebrospinal fluid
CTCs	circulating tumor cells
EDTA	ethylene-diamine-tetra-acetic acid
ER	estrogen receptor
FFPE	formalin fixed paraffin embedded
Her2 (or ErbB2)	human epidermal growth factor receptor-2
IBC	inflammatory breast cancer
MMR	mismatch repair
NGS	next generation sequencing
OCT	optimal cutting temperature compound
OR	odds ratios
OS	overall survival
PR	progesterone receptor
RBC	red blood cells
SNVs	single nucleotide variants
TN	triple negative
VCF	variant call format
