# Supplementary material for: Genetic Variants Detected Using Cell-Free DNA from Blood and Tumor Samples in Patients with Inflammatory Breast Cancer
Source: Int J Mol Sci. 2020 Feb 14;21(4):1290. doi: 10.3390/ijms21041290 (PMC7072950; doi:10.3390/ijms21041290)
Supplement: Supplementary file 1 [file ijms-21-01290-s001.zip › ijms-703397 SI/ijms-703397 SI Data.doc]

**Supplementary Data: Patient clinical history**

**Triple negative IBC Group**

**Patient 1 (T77549)-** The patient had invasive ductal carcinoma (IDC) triple negative in the right breast two years before IBC diagnosis; 5/11 positive lymph nodes.

Month 0: IBC diagnosed in right breast; stage IIIC; triple negative. Month 1: bilateral simple mastectomy; no evidence of carcinoma in the left breast. Month 2–9: adjuvant chemotherapy docetaxel (Taxotere) and carboplatin for eight cycles. Month 10: patient developed chest wall recurrence and started capecitabine (Xeloda). Month 11: PET/CT scan showed increased activity in small left axillary and internal mammary lymph nodes; increased size of left axillary lymph node. Month 12: patient presented to Fox Chase Cancer Center (FCCC) for treatment recommendations; erythema extending from chest wall to the upper abdomen skin (stage IV); started ixabepilone (Ixempra); right inguineal lymph node and plasma collected for future next generation sequencing (NGS). Month 13: ixabepilone stopped due to increase activity on the lung. Month 15: started epirubicin. Month 17: epirubicin stopped due to disease progression; erythema on her chest wall continued to extend; there was some progression in the activity of the left axilla and left upper chest wall, and the erythema on the left chest wall continued to increase. Month 18: started phase 1 clinical trial AZD1480 Jak2 inhibitor; shortly after stopped due to disease progression. Month 18–19: started doxorubicin (Doxil) and cyclophosphamide (Cytoxan). Month 20: PET/CT scan detected brain metastases and increased activity in lymph nodes and chest wall. Month 20–21: started paclitaxel (Abraxane) and gemcitabine. Month 22: PET/CT scan showed bone, liver, brain metastases; stopped paclitaxel, and gemcitabine; plasma collected for future next generation sequencing (NGS). Month 22–24: began cisplatin and lapatinib (Tykerb); stopped due to disease progression. Month 24–25: Metformin; stopped due disease progression. Month 27: patient died.

FAMILY HISTORY: Two first-degree relatives had breast cancer (one with onset at 52- and another not register) and another first-degree relative (father) had colon cancer. Second-degree relative from mother side had breast cancer (onset at 69), and from the father’s side, one relative with breast cancer at age 41.

**Patient 2 (C65525)-** Month 0: IBC diagnosed in left breast; stage IIIB; T4d N1 M0; triple negative. Month 1–5: neoadjuvant doxorubicin (Adriamycin) and cyclophosphamide for four cycles followed by one cycle of docetaxel (Taxotere) with a significant bad reaction; patient was switched to paclitaxel (Taxol). Month 7: left mastectomy with persistent extensive dermal lymphatic invasion with high-grade ductal involvement and 11/13 positive lymph nodes. Month 8–9: radiation therapy. Month 11: patient presented to Fox Chase Cancer Center; no clinical evidence of disease; PET scan normal. Month 14–15: developed local chest wall recurrence; skin rash that began in the right breast and extended to the chest wall of the previous surgery; biopsy proved to be recurrent IBC (stage IV). Month 15–20: carboplatin and gemcitabine for 4 cycles; stopped due to skin disease progression; second chest wall recurrence. Month 20–21: capecitabine; stopped due to progressive disease in the chest wall. Month 21–24: started vinorelbine (Navelbine); stopped due to skin disease progression. Month 24–26: started ixabepilone (Ixempra); stopped due to skin disease progression; the skin changes extended to the contralateral breast and the posterior left chest wall with diffuse redness; no ulceration; PET scan showed no bone or other site metastases. Month 27: doxorubicin (Doxil) for one cycle while awaiting eligibility for phase I clinical trial. Month 27–28: skin disease progression with pruritus; started bevacizumab (Avastin; angiogenesis inhibitor; vascular endothelial growth factor A (VEGF-A) inhibitor); stopped due to skin disease progression; left IBC with recurrent disease to the skin and right axillary lymph nodes (stage IV). Month 28–30: started clinical trial with ALK inhibitor; on month 29, improvement in the skin of the left chest wall, left back and right breast; CT scan showed stable disease (month 29). Month 30: skin disease progression, with lesions covering a larger surface area; left inflammatory breast cancer with recurrent disease to the skin and right axillary lymph nodes; stopped ALK inhibitor due to skin disease progression. Month 30–33: started cyclophosphamide (Cytoxan), methotrexate and 5-fluorouracil (5-FU); stopped on month 33 due to skin disease progression; serum and breast tissue biopsy collected for future NGS (month 33). Month 34: PET scan showed bone metastases; spine metastases; minimal pleural fluid. Month 35: started compassionate treatment with gene therapy SGT-P53; stopped due to delirium; MRI revealed multiple brain metastases; whole brain radiation; metastatic disease to mediastinum, right axilla, and pleura and extensive chest wall disease. Month 38: Patient died.

FAMILY HISTORY: First-degree relative had lung cancer and was a heavy smoker.

**Patient 3 (L67523)-** Month 0: IBC of the left breast, triple negative, no metastases in either abdomen, pelvis or bone. Month 1–4: neoadjuvant chemotherapy with doxorubicin (Adriamycin) and cyclophosphamide (Cytoxan) four cycles followed by four cycles of paclitaxel (Taxol). Month 5: the patient came to FCCC for a second opinion; she had clinical residual disease; T4dN3M0; patient had a minimal response to the neoadjuvant chemotherapy with persistent skin thickening, breast residual disease and multiple lymph nodes in the area as demonstrated by the PET/CT. Month 5–7: started combination of carboplatin and gemcitabine with Neulasta support; four cycles with some initial response but lately with minimal improvement. Month 7–8: started neoadjuvant radiation therapy with capecitabine (Xeloda); showed clinical response. Month 10: left breast mastectomy and axillary lymph node dissection; patient had residual disease at the time of surgery; 21/21 lymph nodes were positive. Month 11–12: started chemotherapy with capecitabine (Xeloda); developed pleural effusions positive for tumor cells; serum and cells from malignant pleural effusion were collected and stored for future NGS (month 12). Month 13–15: started ixabepilone (Ixempra), paclitaxel (Abraxane) and bevacizumab (VEGF-A inhibitor) with Neulasta support; recurrent metastatic IBC to the pleura, mediastinum, and contralateral disease; showed evidence of response; stopped bevacizumab due to skin infection; stopped due to disease progression. Month 16–17: IBC of the left breast, with recurrence to the left chest wall, pleural fluid, bone, and supraclavicular, mediastinal and right axillary lymph nodes; clinical trial phase I, Multicenter, Open-Label Dose Escalation Study of LDK378, administered orally in adult patients with tumors characterized by genetic abnormalities in anaplastic lymphoma kinase (ALK). Month 17: developed ascites; patient died.

FAMILY HISTORY: patient’s mother had colon cancer (onset at 72-years old); second-degree relative from mother side had colon cancer (onset at 62-years old). Second-degree relatives from father side had melanoma (age 70) and another relative had also colon cancer.

**Patient 4 (I74311)-** Month 0: IBC in the left breast, triple negative. Month 1–7: neoadjuvant therapy: phase 2 trial NSABP-FB6, using doxorubicin (Adriamycin) and cyclophosphamide (Cytoxan) followed by weekly paclitaxel plus pazopanib (pazopanib is a multi-targeted receptor tyrosine kinase inhibitor); showed clinical response; patient came off study on month 7 due to significant neutropenia, completing 11 cycles of paclitaxel. Month 9: left modified radical mastectomy, left axillary lymph node dissection, right prophylactic total mastectomy, placement of bilateral breast tissue expanders, and laparoscopic bilateral salpingo- oophorectomy; bilateral mastectomy since she had a *BRCA1* mutation; 11/14 positive lymph nodes; stage III. Month 10–11: six weeks of standard radiation. Month 16: erythema of the skin with nodularity was noted; chest wall recurrence from previous IBC; lung metastases. Month 17–19: she came to FCCC for treatment recommendation; skin breast biopsy and serum collected for future NGS analysis (month 17); started phase I trial with combination of doxorubicin (Adriamycin), cyclophosphamide (Cytoxan) with ABT-888 (PARP inhibitor) cycle 1 and 2; stopped due to progressive disease. Month 20–23: gemcitabine and carboplatin; stopped due to progression in the skin of the chest wall. Month 24–25: eribulin; patient progressed after 4 cycles. Month 26-30: ixabepilone in combination with capecitabine. Month 31–41: not known. Month 42: patient died.

Family history: patient’s father had prostate cancer at the age of 64. Second-degree relatives from father side had breast cancer at age 38 and another relative had breast cancer at the age of 43 and ovarian cancer at the age of 47 and died of her disease. A germline *BRCA1* mutation was detected in the patient by a clinical genetic test.

**Patient 5 (K75070)-** Month 0**:** IBC stage IIIC (T4d N3 M0), triple negative carcinoma grade 3 of the right breast; also, stage I (T1N0M0) invasive lobular ER+PR+Her2- carcinoma with DCIS on the left breast. Month 1–7: patient presented to Fox Chase Cancer Center for treatment recommendations; skin breast biopsy and plasma collected and stored for NGS; start neoadjuvant chemotherapy using 5-fluorouracil with epirubicin for 4 cycles followed by 12 weeks of weekly paclitaxel (Taxol). Month 8: completed pathological response; right modified radical mastectomy and left simple mastectomy with sentinel node biopsy. Month 9–10: adjuvant radiation. Month 15: recurrent IBC; parenchymal brain metastases and leptomeningeal carcinoma. Month 17: patient died.

FAMILY HISTORY: patient’s mother had breast cancer at the age of 65. Second-degree relatives from mother’s side had two relatives that had breast cancer, one apparently with CNS brain metastases (spinal and brain). Maternal second cousin with breast and thyroid cancer. Other maternal third-degree relatives with breast, thyroid, lung, rectal, and colon cancers.

**Patient 6 (B79071)-** Month 1: IBC of the right breast, T4dN3M1 with contralateral lymph nodes at the time of initial diagnosis; triple negative. Month 3–6: neoadjuvant therapy with doxorubicin (Adriamycin) and cyclophosphamide (Cytoxan) and paclitaxel (Taxol). Month 8: mastectomy with immediate reconstruction; extensive residual disease, lymph node positive. Month 26: recurrence of the chest wall and progression in the contralateral lymph nodes. Month 27–34: treated with two different lines of therapy including gemcitabine (Gemzar) and carboplatin apparently with some response and after vinorelbine and bevacizumab (Avastin; VEGF-A inhibitor) with clinically progressive disease. Month 35: patient came to FCCC for treatment recommendations; no brain metastases; no bone metastases; liver metastases; progressing disease on the chest wall and liver; skin breast biopsy and serum collected and stored for NGS analysis (month 35). Month 36–49: patient transferred to another institution; no data available. Month 50: pleural effusions; patient died.

FAMILY HISTORY: Patient’s mother had cervical cancer in her 30s and a first-degree relative from her mother’s side had breast cancer at the age of 47. Second-degree relative from mother’s side had lung cancer.

**Patient 7 (S80274)-** Month 0: IBC diagnosed in the right breast; stage IIIC; T4d N3 M0; triple negative. Month 1–4: doxorubicin (Adriamycin) and cyclophosphamide (Cytoxan) for four cycles followed by paclitaxel (Taxol); stopped for neuropathy. Month 5: right modified radical mastectomy; 10/10 positive lymph nodes. Month 6–8: radiation therapy. Month 11–12: capecitabine (Xeloda); stopped due to disease progression; two sites of disease recurrence, the skin and contralateral axillary lymph node (stage IV), without any other distant metastasis; skin disease progression to the right arm. Month 13: patient presented to Fox Chase Cancer Center for treatment recommendations; PET/CT scan and brain MRI showed no metastatic disease other than the skin and contralateral axillary lymph node; skin breast biopsy and serum collected for NGS (month 13). Month 14–17: ixabepilone (Ixempra) for five cycles; stopped on month 17 due to progressive disease in the skin; the erythema on her right chest wall continued to spread to the right upper quadrant of her abdomen and to the right mid-axillary line. Month 18: started gemcitabine and carboplatin; stopped after two cycles for progression skin disease and new mediastinal lymph nodes. Month 20–21: paclitaxel (Abraxane) for two cycles, with lapatinib added with second cycle; stopped for progression of disease in the skin, left breast and left axilla. Month 21–24: started docetaxel, lapatinib (Tykerb); pleural effusions. Month 24: metastatic IBC confined only to the chest wall with minimal progression on PET/CT scan. Month 25–26: began a phase I study of the anti-A5B1 integrin monoclonal antibody, PF-04605412; stopped due to disease progression; developed pleural effusions. Month 26: started eribulin/ Month 30: patient started cyclophosphamide (Cytoxan), methotrexate and 5-fluorouracil (5-FU); CT chest revealed evidence of progressive disease on the chest wall and right axillary adenopathy; possibility of right chest wall posterolateral right lung involvement; no evidence of liver disease; the MRI of the brain was negative. Month 31: patient died

Family history: No family history of cancer.

**Patient 8 (K93878)-** Month 0: IBC in left breast; stage III; triple negative. Month 3–6: neoadjuvant chemotherapy with docetaxel (Taxotere), doxorubicin (Adriamycin) and cyclophosphamide for six cycles. Month 7: bilateral mastectomy with immediate reconstruction followed by radiation to the left side. Month 25: metastatic carcinoma to the right side and bone. Month 26–32: paclitaxel (Taxol) with Zometa for bone disease. Month 32–37: started capecitabine (Xeloda); increased bone metastasis especially in the ribs and hips; radiation therapy. Month 37–40: ixabepilone (Ixempra) with Neulasta support, completed 6 cycles; stopped due to progression. Month 41: started gemcitabine and denosumab (Xgeva; to treat bone metastases); developed malignant pleural effusions. Month 43–44: doxorubicin (Adriamycin) and denosumab (Xgeva). Month 44: patient came to Fox Chase Cancer Center for treatment recommendation; metastatic IBC with metastases to the bone, bladder, chest wall, malignant pleural effusion and pelvic ascites; cells from malignant pleural effusion and serum were collected for future NGS (month 44); patient died

FAMILY HISTORY: From her mother’s side, first-degree relatives had endometrial cancer at young age, pancreatic cancer (onset at 78), and prostate cancer (onset at 61). A third degree relative from her mother’s side had breast cancer. A second-degree relative from her father’s side had colon cancer.

**Patient 9 (D89802)-** The patient had triple negative invasive ductal carcinoma (IDC) in the right breast seventeen months prior to IBC diagnosis; treated with docetaxel (Taxotere), doxorubicin (Adriamycin) and cyclophosphamide; bilateral mastectomy with right axillary lymph node dissection and implants for breast reconstruction; clinical trial randomized to receive cisplatin with PARP inhibitor for 4 cycles followed by an oral PARP inhibitor.

Month 0: IBC diagnosed in right reconstructed breast with positive lymph nodes in the right chest wall and right axilla and a lung lesion in the left upper lobule (stage IV); triple negative. Month 1: patient presented to Fox Chase Cancer Center for treatment recommendations. Month 1–2: paclitaxel (Abraxane) for two cycles; progression in the right axillary, bilateral supraclavicular and hilar lymphadenopathy and in the skin of the right reconstructed breast. Month 3–8: extensive skin disease on the right breast with ulcerations; breast tissue biopsy and serum collected for NGS (month 3); started ixabepilone (Ixempra) and capecitabine (Xeloda). Month 8: Ixempra and Xeloda stopped due to skin disease progression. Month 8–9: started carboplatin and gemcitabine (Gemzar). Month 10–19: patient transferred to another institution; no data available (NDA). Month 19: patient died.

FAMILY HISTORY: Patient’s father had bladder cancer (onset at 68). Paternal second-degree relatives had pancreatic cancer and a third-degree relatives had breast cancer at age 87. Maternal second-degree relative had colon cancer (onset at age 70). A germline *BRCA2* mutation was detected in the patient by a clinical genetic test.

**Patient 18 (E78569)-** Month 0: Lump in the left breast; infiltrating ductal carcinoma, grade 3, triple negative; T3N1M0. Month 1–4: received neoadjuvant chemotherapy doxorubicin (Adriamycin) and cyclophosphamide (Cytoxan) for 4 cycles followed by paclitaxel (Taxol) for 12 cycles. Month 5: lumpectomy showed invasive ductal carcinoma triple negative, as well as DCIS. Month 6: bilateral total skin sparing mastectomy with immediate reconstruction with tissue expanders; 3/18 positive lymph nodes. Month 8–9: radiation therapy for six doses. Month 10–15: standard follow-up. Month 16: the patient developed pericardial and bilateral pleural effusions, right para-tracheal and right axillary lympho-adenopathy; evidence of recurrence. Month 16–18: started doxorubicin (Doxil) and ABT-888 PARP inhibitor as phase I on a weekly dose; stopped due to disease progression to the left supraclavicular region and posterior chest. Month 20: started clinical trial with cisplatin and RAD001 (everolimus); stopped due to disease progression. Month 21: first time at FCCC; recurrent IBC on the left breast with recurrence to the chest wall, pleura and pelvis; aggressive advanced disease; no evidence of brain metastases; MRI could not be performed due to tissue expanders in place; plasma collected for future NGS analysis (month 21); started carboplatin and gemcitabine. Month 22–25: started ixabepilone (Ixempra) and capecitabine (Xeloda); stopped Ixempra and Xeloda due to disease progression. Month 26: disease localized in pleura; chest wall had multiple skin nodules and pericardial effusion and lymph nodal disease within the chest and abdomen; T4dN3M; cells from malignant pleural effusion collected and stored for future NGS analysis (month 26); started eribulin and denosumab. Month 27: progressed on eribulin and denosumab; disease involving skin, pleural disease and pericardial disease as well as chest parenchyma; CTCs= 143; patient died.

FAMILY HISTORY: Third-degree relative had breast cancer at the age of 56.

**Patient 19 (S73507)-** Month 0**:** patient self-palpated a mass in the upper part of the right breast with minimal skin changes. Month 7: infiltrating ductal carcinoma, grade 3, ER/PR and HER-2/neu negative; IBC on the right breast. Month 8: patient came to FCCC; PET/CT showed extensive hypermetabolic disease in multiple locoregional lymph nodes and mediastinal and hilar lymph nodes with some concern for marrow involvement; metastatic disease to the mediastinal lymph nodes and possible bone; stage IV (T4dN3M1). Month 8–13: skin breast biopsy collected and stored for future NGS (month 8); neoadjuvant weekly paclitaxel (Taxol) and, 5 fluorouracil (5FU) and epirubicin for six cycles; completed with a complete clinical response. Month 15: right modified radical mastectomy; plasma collected for future NGS (Month 15). Month 18: radiation therapy and capecitabine (Xeloda); no clinical evidence of progressive disease. Month 21: continue on Xeloda. Month 39: breast reconstruction. Month 47: no evidence of any additional recurrence of disease; last record at FCCC.

FAMILY HISTORY: Second-degree relative from father’s side had colon cancer (onset at 60-years old). Five maternal third-degree relatives had breast cancer.

**ER-positive Her2-negative IBC**

**Patient 10 (M71182)-** The patient had ER+ (10%) PR+ (30%) Her-2 invasive ductal carcinoma (IDC) in the left breast one year before IBC diagnosis; patient underwent bilateral mastectomy with implants and reconstruction; 0/7 positive lymph nodes.

Month 0: IBC in left breast; stage IIIB; ER+ (10%) PR+ Her2-; skin nodules and chest wall recurrence on the surgical scar on the left side of the reconstructed breast. Month 1–3: docetaxel (Taxotere), doxorubicin (Adriamycin) and cyclophosphamide (Cytoxan) for three cycles. Month 4–13: additional disease within the surgical area; underwent DP flap, left breast removal of implant, standard radiation concomitant with carboplatin completed on month 8, and then received vinorelbine (Navelbine) for 12 weeks. Month 14: started Tamoxifen; normal PET/CT scans until month 26. Month 26–29: lymph nodes in the internal mammary area were identified with a PET/CT, and suspicious area in the left lung. Month 30: patient developed another recurrence in chest wall. Month 31–35: started on paclitaxel (Abraxane) and bevacizumab (Avastin; VEGF-A inhibitor) for six cycles. Month 38: developed more nodules and rash; treated with antibiotics and letrozole (Femara; aromatase inhibitor); biopsy ER+ (28%) Her2 negative. Month 40–43: photo-dynamic therapy/ Month 54: patient came to Fox Chase Cancer Center for treatments recommendations; recurrent metastatic IBC stage IV; disease in the chest wall, the mediastinum, lymph nodes, liver and bone; no brain metastases; disease was progressing under current treatment with Femara; skin breast biopsy and plasma collected for future NGS studies (month 54). Month 54–59: capecitabine (Xeloda) and Zometa; stopped Xeloda due to disease progression. Month 60: increase in the ulceration of the chest wall. Month 60–66: ixabepilone (Ixempra) and denosumab (Xgeva; for bone metastases); stopped due to disease progression in liver. Month 66–69: started eribulin; stopped due to disease progression; significant erythema and skin thickening with nodularity, progression of disease in the skin, liver, bone, and lymph nodes. Month 70–71: denosumab ( Xgeva). Month 71–75: started on Afinitor (mTOR inhibitor) and exemestane (Aromasin; aromatase inhibitor); PET-CT showed significant progression of the disease in liver; stopped treatment and went to hospice care. Month 76: extensive chest wall disease with liver and bone metastases; patient died.

FAMILY HISTORY: Second-degree relatives had melanoma at the age of 70, spinal cancer at 67-years old and colon cancer at 62-years old; a third-degree relative had pancreatic cancer at age 83.

**Patient 11 (M85099)-** Month 0: IBC in right breast; stage IIIB; T4dN0M0; ER+Her2-. Month 2–5: neoadjuvant chemotherapy with docetaxel (Taxotere), doxorubicin (Adriamycin), and cyclophosphamide for six cycles. Month 6: right breast mastectomy. Month 7–9: adjuvant radiation therapy. Month 9–18: adjuvant therapy with tamoxifen (endocrine therapy). Month 14: developed metastasis in lung (stage IV). Month 18: patient came to Fox Chase Cancer Center for treatments recommendations; metastases in lungs, lymph nodes, bones and left pleural effusion; no brain metastasis. Month 18–25: started ixabepilone (Ixempra) and capecitabine (Xeloda); completed with excellent response by imagen and patient became asymptomatic by month 25. Month 26–28: discontinued Ixempra and continued Xeloda, and started with anastrazole (Arimidex; aromatase inhibitor) and Zometa for bone disease. Month 28: discontinued Xeloda, and continued with Arimidex as single agent and Zometa. Month 29: PET scan showed evidence of progressive disease; increase pleural fluid with metabolically active pleural disease; developed also metastatic disease in liver; multiple active lymph nodes in the upper abdomen possibly related to metastatic disease; cells from malignant pleural effusion and serum collected for future NGS (month 29). Month 30–32: started second line endocrine therapy everolimus (Afinitor; mTOR inhibitor) and exemestane (Aromasin; aromatase inhibitor), along with denosumab for bone metastases (Xgeva); stopped due to disease progression mainly in the liver. Month 32–36: changed from combination of endocrine therapy to chemotherapy with carboplatin and gemcitabine (Gemzar); stopped due to disease progression. Month 36–41: started eribulin; stopped due to disease progression. Month 41–44: started doxorubicin (Doxil); stopped due to disease progression. Month 44–46: started ixabepilone (Ixempra); stopped due to disease progression. Month 47: started clinical trial. Month 50: metastatic disease in lungs, lymph nodes, bones and liver; patient died.

FAMILY HISTORY: Second-degree relatives had lung cancer and prostate cancer.

**Patient 12 (P73793)-** Month 0: Self-palpated a mass and noted pain in the left axilla. Month 1: left IBC stage IIIB T4dN1M0, ER+PR+Her-2/Neu negative; breast tissue biopsy and serum collected for NGS studies. Month 1–7: paclitaxel for 12 weekly doses followed by 5-FU, cyclophosphamide and epirubicin for six cycles. Month 8: left modified radical mastectomy, showed no residual carcinoma within the breast, nipple or skin, with metastatic carcinoma in 6/15 lymph nodes. Month 11: hyper-fractionated twice daily radiation therapy for four weeks; started Arimidex. Month 12–56: patient transferred to another institution and no medical records available. Month 57: last record available at FCCC.

FAMILY HISTORY: First-degree relative had CNS lymphoma at the age of 81. Second-degree relatives had breast cancer (at age 60), and another throat cancer (smoker).

**Patient 13 (B68225)-** One year before, the patient had invasive ductal carcinoma ER+PR- HER-2/Neu negative on her right breast.

Month 0-8: IBC on the right breast with chest wall progression; patient had evidence of progressive disease in the right breast and the opposite breast. A left core biopsy was done on the left side. After bilateral mastectomy was done and found to have skin changes to suggest a recurrence secondary to IBC on the right side; she completed radiation. Month 8: chest wall progression and started on bevacizumab (Avastin; VEGF-A inhibitor) and paclitaxel (Taxol) with some improvement over time. One of the areas that progressed during this time was also treated with electron beam radiation to the lateral aspect of the chest. Month 16: patient progressive disease and started eribulin; there were some changes to suggest progression on the chest wall and on the shoulder. Month 19: patient came to FCCC for second opinion; diffuse metastatic IBC (stage IV), mostly localized to the skin of the chest wall and contralateral breast; skin breast biopsy and serum collected for NGS studies; began chemotherapy with capecitabine (Xeloda), gemcitabine (Gemzar) and carboplatin. Month 19–39: patient followed in another hospital; no records available. Month 40: patient died.

FAMILY HISTORY: First-degree relative had acute myeloid leukemia (AML) diagnosed at the age of 72. Second-degree relatives had lymphoma, another relative had kidney cancer (in their 60s). Another first-degree relative had colon cancer (at 77-years old).

**Patient 14 (B78899)** - Patient had ER+PR-Her2- IBC but after became triple negative (at month 10).

Month 0: swelling of the left breast associated with heaviness, redness, rash, and this was also accompanied by an increase in the size of the right side of the body with retention of fluid and increase in approximately 10 lbs of weight. Month 1: palpable axillary node and a core biopsy that revealed invasive lobular carcinoma, grade 2, with extensive lymphovascular invasion. The tumor was ER+PR-Her2-. Month 2–3: because the patient had a previous history of cardiac problems, she was first started on docetaxel (Taxotere) every two weeks for four cycles; she had some improvement, but still significant residual disease. Month 4–5: she was treated with neoadjuvant 5 fluorouracil, epirubicin, and cyclophosphamide three cycles; at the end of the second cycle, the patient developed hypotension and pulmonary edema; she also had a thoracentesis that removed 300 cc from the right lung without any evidence of metastatic disease; diagnosed with cardiac failure. Month 7: mastectomy; extensive infiltrating lobular carcinoma residual with dermal invasion; also contralateral prophylactic mastectomy that was negative. The right mastectomy and axillary dissection revealed 18/19 positive lymph nodes. Month 8: patient came to FCCC for treatment recommendation; skin breast biopsy and plasma collected for future NGS; she had evidence of skin nodules and rash on the right chest wall, and evidence of progressive metastatic disease. CT scan at that time showed not only lymphadenopathy in the mediastinal area which was suspicious at the beginning, and also diffuse abdominal lymph nodes; right upper chest punch biopsy above mastectomy scar showed ER-PR-Her2-. Month 9: PET CT confirmed the presence of extensive chest wall disease, not only on the right side that extends all the way to the axillary line and seems to involve also the muscle; extensive bony metastases and the most hypermetabolic activity was present at the level of the rib cage on the right side, but she had multiple vertebral bodies including T-spine, L5, and also in the pelvic area; also had pleural fluid on the right; CT of the brain without contrast showed that there was no gross evidence of brain metastasis; proposed a combination of Ixempra and Xeloda. Month 10: patient died.

FAMILY HISTORY: First-degree relative had prostate cancer at age 79 with prostate cancer. Second-degree relative had lung cancer. Patient had Ehlers–Danlos syndrome.

**Patient 16 (T73616) -** IBC of the right breast, invasive ductal carcinoma, ER+PR+HER-2 negative. Month 3: patient comes to FCCC; plasma collected and stored for future NGS analyses. Month 4: started paclitaxel for four weeks followed by FED; MRI showed widespread metastatic disease with involvement of every vertebral body; IBC stage IV (T4d N1 M1) with metastases to the bone; MRI of the brain without evidence of metastatic disease; Zometa for bone disease. Month 5: paclitaxel stopped due to plateau response and toxicity; started 5-FU, epirubicin and cyclophosphamide every three weeks with Neulasta support. Month 6: started docetaxel (Taxotere) and cyclophosphamide every three weeks with Zometa every six weeks. Month 8: IBC metastatic to the bone and pleural fluid. Month 9: a liver biopsy was consistent with metastatic adenocarcinoma; IBC of the right breast, ER/PR positive, HER-2 negative, with metastatic disease to the bone, pleural fluid, bone marrow, and liver; cells from malignant pleural effusion collected for future NGS (month 9); started capecitabine (Xeloda). Month 10: MRI of the brain revealed no metastatic disease to the brain parenchyma. Month 11: stable disease; continued with Xeloda and added fulvestrant (Faslodex; endocrine therapy) for progressive disease in the bone and bone marrow. Month 14: on treatment with Faslodex with Xeloda, and monthly denosumab. Month 16–17: radiation therapy to the lumbar spine. Month 18–27: continue with Faslodex, Xeloda and denosumab. Month 28–39: no information available. Month 40: Faslodex and Xgeva monthly; the patient was asymptomatic. Month 41: patient stopped Xeloda; continue on Faslodex and denosumab (Xgeva; for bone metastases); disease continue in remission. Month 42–61: continue on Faslodex and Xgeva; disease in stable remission. Month 62: last record available

FAMILY HISTORY: First-degree relative from patient mother’s side had breast cancer at the age of 40 and survived. Three second-degree relative from patient mother’s side had breast cancer (one at age of 60).

**ER-positive Her2-positive IBC**

**Patient 15 (B62630)-** Month 0: IBC in left breast; ER+PR+Her2+; stage III. Month 6–10: neoadjuvant therapy with doxorubicin (Adriamycin) and cyclophosphamide every two weeks for two cycles with no response; docetaxel (Taxotere), doxorubicin (Adriamycin) and cyclophosphamide for 4 cycles; stopped for the develop of contralateral disease. Month 10–11: capecitabine (Xeloda); radiation/ Month 12: bilateral mastectomy; 8/13 positive lymph nodes. Month 14–21: started Herceptin, tamoxifen and Zometa for bone disease. Month 22: disease recurrence in skin. Month 23–29: lapatinib (Tykerb) and capecitabine (Xeloda); stopped due to disease progression in the skin. Month 32–41: goserelin (Zoladex; gonadotropin releasing hormone agonist) and letrozole (Femara; aromatase inhibitor); patient did not receive chemotherapy. Month 42: disease progression with new pleural effusions. Month 42–44: Zoladex and fulvestrant (Faslodex). Month 44–46: Zoladex; progression in the skin, pleural effusion and mediastinal lymph nodes. Month 46–49: patient discontinued all treatment; continued to have progression in the skin and pleural effusions. Month 49: patient presented to Fox Chase Cancer Center for treatment recommendations; metastatic IBC to the skin and pleura (stage IV); PET/CT scan showed hypermetabolic mediastinal and bilateral lymph nodes, evidence of multiple areas of disease, pleural thickening in the left side, and also chest wall disease; tumor was ER+PR-Her2-. Month 50: cells from pleural effusion and serum collected for NGS; ixabepilone (Ixempra) for two cycles; stopped due to allergic reaction after second cycle and progression of the disease in the skin and lungs with lymphangitic spread. Month 51–53: started carboplatin, Herceptin and lapatinib (Tykerb); on month 53, stopped due to disease progression in the skin. Month 54–55: metastases in right lung; radiation therapy. Month 56: metastatic left IBC with disease in the right breast, skin, lymph nodes and pleura; started with doxorubicin (Doxil); patient died.

FAMILY HISTORY: A third-degree relative had breast cancer and died at the age of 39.

**ER-negative Her2-positive IBC**

**Patient 17 (S69308)-** Month 0: Invasive ductal carcinoma of the right breast (probably T3N1M0) ER-PR-Her2+ (2+). Month 1–5: neoadjuvant therapy with doxorubicin (Adriamycin) and cyclophosphamide followed by paclitaxel (Taxol) and Herceptin. Month 6: right modified mastectomy; two positive lymph nodes. Month 7–8: radiation therapy and Herceptin. Month 9–15: Herceptin. Month 21: skin rash; recurrent IBC. Month 22–24: started Herceptin plus vinorelbine (Navelvine); stopped due to low tolerance. Month 25–27: plasma collected for NGS (month 25); started capecitabine (Xeloda) and lapatinib (dual tyrosine kinase inhibitor which interrupts the HER2/neu and EGFR pathways). Month 27: patient comes to FCCC for evaluation; IBC with chest wall disease, lymph nodes in the supraclavicular region on the left as well as axillary region; no evidence of brain metastases; started Herceptin and carboplatin. Month 29–31: nab-paclitaxel (Abraxane) with carboplatin; skin breast biopsy and plasma collected for future NGS. Month 32: progressive disease; she discontinued chemotherapy; minimal pleural effusion accumulating in the right side. Month 33–37: no records available. Month 37: patient died.

Patient medical history: Sjogren's syndrome

Family history: Second-degree relative had kidney cancer.
