# Supplementary material for: Genetic Variants Detected Using Cell-Free DNA from Blood and Tumor Samples in Patients with Inflammatory Breast Cancer
Source: Int J Mol Sci. 2020 Feb 14;21(4):1290. doi: 10.3390/ijms21041290 (PMC7072950; doi:10.3390/ijms21041290)
Supplement: Supplementary file 1 [file ijms-21-01290-s001.zip › ijms-703397 SI/Supplementary Legends for Table 3 and Table 4.doc]

**Supplementary Table 3 and Table 4 Legends**

**Supplementary Table 3. Genetic variants detected in all the samples of the 19 IBC patients.** A total of 416 variants were detected in all studied samples. A total of 37 false positives were detected which are indicated in the last column of the table as “1” (Called Artifact). Variants from cfDNA and/or tumor tissue or cells from 19 IBC patients are shown. SeraCare Controls 0.25%, Control 0.5%, Control 1%, Control 2% and wild type (Control WT) are also shown. For patient 12 (P73793), one tissue sample (month 1) and two samples of cfDNA (month 1) were studied: cfDNA1 (plasma; month 1) and cfDNA2 (serum; month 1); although only cfDNA2 was used for further analysis as the quantity of cfDNA to prepare the NGS library was greater. For patient 1 (T77549), one tissue sample (month 12) and two samples of cfDNA were run: cfDNA1 (plasma; month 12) and cfDNA2 (plasma; month 22); only data from cfDNA1 was used for further analysis since its collection was done at the same time as the tissue sample. Variants were filtered out if their allele fraction (AF) was lower than 2% for tissue or malignant cells, or lower than 1.5% for cfDNA from plasma or serum. Synonymous variants were also filtered out and any exonic and/or splicing variant were retained (this excluded intronic events, but also events labeled intergenic, associated with 3’ or 5’ UTR).

**Supplementary Table 4. Genetic variants detected in 15 patients with matched blood cfDNA and tissue samples collected at the same time.** The time of collection of blood matched the time of collection of the tissue in 15 patients: patient 1 (T77549), patient 2 (C65525), patient 3 (L67523), patient 4 (I74311), patient 5 (K75070), patient 6 (B79071), patient 7 (S80274), patient 8 (K93878), patient 9 (D89802), patient 10 (M71182), patient 11 (M85099), patient 12 (P73793), patient 13 (B68225), patient 14 (B78899) and patient 15 (B62630). Data from these patients were extracted from Supplementary Table 3. Germline variants observed in the overall population with an allele frequency of 2.5% or greater in the Genome Aggregation Database (gnomAD) were excluded from the data.
