# Supplementary material for: Genetic Variants Detected Using Cell-Free DNA from Blood and Tumor Samples in Patients with Inflammatory Breast Cancer
Source: Int J Mol Sci. 2020 Feb 14;21(4):1290. doi: 10.3390/ijms21041290 (PMC7072950; doi:10.3390/ijms21041290)
Supplement: Supplementary file 1 [file ijms-21-01290-s001.zip › ijms-703397 SI/Supplementary Table 1.doc]

| **Patient ID** | **IBC subtype** | **Age at diagnosis** | **Race** | **Overall Survival**  **(OS)** |
| --- | --- | --- | --- | --- |
| Patient 1  (T77549) | TN | 47 | White | 27 months |
| Patient 2  (C65525) | TN | 55 | White | 38 months |
| Patient 3  (L67523) | TN | 46 | White | 17 months |
| Patient 4 (I74311) | TN | 43 | White  (Ashkenazi Jewish heritage) | 42 months |
| Patient 5  (K75070) | TN | 43 | White | 17 months |
| Patient 6  (B79071) | TN | 48 | African-American | 50 months |
| Patient 7  (S80274) | TN | 61 | White | 31 months |
| Patient 8  (K93878) | TN | 43 | African- American | 44 months |
| Patient 9  (D89802) | TN | 44 | White | 19 months |
| Patient 10  (M71182) | ER+ Her2- | 34 | White | 76 months |
| Patient 11  (M85099) | ER+ Her2- | 44 | White | 50 months |
| Patient 12  (P73793) | ER+Her2- | 57 | White | 57 months  (1) |
| Patient 13  (B68225) | ER+Her2- | 49 | White | 40 months |
| Patient 14  (B78899) | ER+ Her2-  (TN at month 8) | 66 | White  (Jewish heritage) | 10 months |
| Patient 15  (B62630) | ER+Her2+  (ER+Her2- at month 49) | 37 | Hispanic | 56 months |
| Patient 16  (T73616) | ER+Her2- | 39 | White | 62 months  (1) |
| Patient 17  (S69308) | ER- Her2+ | 69 | Asian | 37 months |
| Patient 18  (E78569) | TN | 48 | White | 27 months |
| Patient 19  (S73507) | TN | 32 | White | 47 months  (1) |

**Supplementary Table 1**. **Patients characteristics.** The age of the patient at the onset of the disease is indicated; (1) last record available. Abbreviations: ER, estrogen receptor; Her2, human epidermal growth factor receptor 2; TN, triple negative
