# Supplementary material for: Genetic Variants Detected Using Cell-Free DNA from Blood and Tumor Samples in Patients with Inflammatory Breast Cancer
Source: Int J Mol Sci. 2020 Feb 14;21(4):1290. doi: 10.3390/ijms21041290 (PMC7072950; doi:10.3390/ijms21041290)
Supplement: Supplementary file 1 [file ijms-21-01290-s001.zip › ijms-703397 SI/Supplementary Table 2.doc]

| ACVR1B | CBFB | ERCC4 | KMT2C | NCOR1 | RAD51 | XRCC2 |
| --- | --- | --- | --- | --- | --- | --- |
| AKT1 | CCND1 | ESR1 | KRAS | NEK2 | RAD51C | XRCC3 |
| APC | CDH1 | EXOC2 | MAP2K4 | NF1 | RAD51D | ZBED4 |
| AR | CDK4 | EXT2 | MAP3K1 | PALB2 | RB1 |  |
| ATM | CDK6 | FAM175A | MDM2 | PALLD | RET |  |
| ATR | CDKN2A | FANCC | MED12 | PBRM1 | SEPT9 |  |
| AXIN2 | CHEK2 | FBXO32 | MEN1 | PCGF2 | SMAD4 |  |
| BAP1 | CSMD1 | FGFR1 | MLH1 | PIK3CA | SMARCA4 |  |
| BARD1 | CTNNB1 | FGFR2 | MRE11A | PIK3R1 | STK11 |  |
| BLM | DIRAS3 | GATA3 | MSH2 | PMS1 | SYNE1 |  |
| BMPR1A | EGFR | GEN1 | MSH6 | PMS2 | TGFB1 |  |
| BRCA1 | EP300 | HERC1 | MUC16 | PPM1L | TP53 |  |
| BRCA2 | EPCAM | HOXB13 | MUTYH | PTEN | TRAF5 |  |
| BRIP1 | ERBB2 | IRAK4 | MYC | PTGFR | VHL |  |
| CASP8 | ERBB3 | ITCH | NBN | RAD50 | WEE1 |  |

**Supplementary Table 2. Breast cancer panel DHS-001Z (Qiagen).** The breast cancer panel used in this study covers 93 genes and is able to detect single nucleotide variants (SNV) and small insertions and deletions (indels). Copy number changes were not studied.
