# Supplementary material for: Genetic Variants Detected Using Cell-Free DNA from Blood and Tumor Samples in Patients with Inflammatory Breast Cancer
Source: Int J Mol Sci. 2020 Feb 14;21(4):1290. doi: 10.3390/ijms21041290 (PMC7072950; doi:10.3390/ijms21041290)
Supplement: Supplementary file 1 [file ijms-21-01290-s001.zip › ijms-703397 SI/Supplementary Table 5.doc]

| Patient  ID | IBC subtype | Total number of  variants | Somatic variants | | | Samples compared |
| --- | --- | --- | --- | --- | --- | --- |
|  |  |  | Number of variants in both cfDNA and tissue | Number of variants only in tissue | Number of variants only in cfDNA |  |
| Patient 1  (T77549) | TN | 17 | 11 | 2 | 4 | Lymph node vs. cfDNA from blood  (month 12) |
| Patient 2  (C65525) | TN | 19 | 12 | 5 | 2 | Breast tissue biopsy vs. cfDNA from blood  (month 33) |
| Patient 3  (L67523) | TN | 13 | 10 | 2 | 1 | Cells from malignant pleural fluid vs. cfDNA from blood  (month 12) |
| Patient 4  (I74311) | TN | 13 | 10 | 3 | 0 | Skin breast biopsy vs. cfDNA from blood (month 17) |
| Patient 5 (K75070) | TN | 10 | 8 | 2 | 0 | Skin breast biopsy vs. cfDNA from blood  (Stage III; month 1) |
| Patient 6  (B79071) | TN | 26 | 20 | 2 | 4 | Skin breast biopsy vs. cfDNA from blood  (month 35) |
| Patient 7  (S80274) | TN | 12 | 7 | 1 | 4 | Skin breast biopsy vs. cfDNA from blood  (month 13) |
| Patient 8  (K93878) | TN | 33 | 25 | 3 | 5 | Cells from malignant pleural fluid vs. cfDNA from blood  (month 44) |
| Patient 9  (D89802) | TN | 9 | 4 | 2 | 3 | Breast tissue biopsy vs. cfDNA from blood  (month 3) |
| Patient 10  (M71182) | Non-TN | 12 | 8 | 4 | 0 | Skin breast biopsy vs. cfDNA from blood  (month 54) |
| Patient 11  (M85099) | Non-TN | 20 | 12 | 6 | 2 | Cells from malignant pleural fluid vs. cfDNA from blood  (month 29) |
| Patient 12  (P73793) | Non-TN | 6 | 3 | 3 | 0 | Breast tissue biopsy vs. cfDNA from blood  (Stage III/ month 1) |
| Patient 13  (B68225) | Non-TN | 10 | 9 | 1 | 0 | Skin breast biopsy vs. cfDNA from blood  (month 19) |
| Patient 14  (B78899) | Non-TN | 9 | 5 | 3 | 1 | Skin breast biopsy vs. cfDNA from blood  (month 8) |
| Patient 15  (B62630) | Non-TN | 12 | 11 | 1 | 0 | Cells from malignant pleural fluid vs. cfDNA from blood (month 50) |
| In all 15 patients |  | **221** | **155** | **40** | **26** |  |

**Supplementary Table 5. Number of variants in tissue and cfDNA in patients in which both samples were collected at the same time.** The variants were detected in: 1) both tissue and cfDNA from blood or, 2) only in tissue or, 3) only in cfDNA from blood. The number of variants detected in each case are indicated. At the time of sample collection, the disease was at Stage IV in all the patients except in patients #5 and # 12 who had Stage III disease.
