# Supplementary material for: Genetic Variants Detected Using Cell-Free DNA from Blood and Tumor Samples in Patients with Inflammatory Breast Cancer
Source: Int J Mol Sci. 2020 Feb 14;21(4):1290. doi: 10.3390/ijms21041290 (PMC7072950; doi:10.3390/ijms21041290)
Supplement: Supplementary file 1 [file ijms-21-01290-s001.zip › ijms-703397 SI/Supplementary Table 6.doc]

| Patient ID | Quantity of cfDNA to prepare NGS library  (cfDNA from blood) | Total number of reads  (cfDNA) | Mean read depth  (cfDNA) | Total number of reads  (tissue/cells) | Mean read depth  (tissue/cells) |
| --- | --- | --- | --- | --- | --- |
| Patient 1  (T77549) | 13 ng (plasma) | 4,438,780 | 1,450.76 | 3,761,410 | 1,270.41 |
| Patient 2  (C65525) | 19 ng (serum) | 3,942,080 | 1,279.20 | 3,754,267 | 1,270.41 |
| Patient 3  (L67523) | 19 ng (serum) | 3,222,292 | 1,073.01 | 3,254,060 | 1,122.18 |
| Patient 4  (I74311) | 30 ng (serum) | 4,987,114 | 718.06 | 7,714,165 | 2,673.43 |
| Patient 5  (K75070) | 16 ng (plasma) | 3,878,882 | 1,303.97 | 7,362,160 | 2,626.14 |
| Patient 6  (B79071) | 23 ng (serum) | 4,993,580 | 1,623.99 | 4,460,607 | 1,435.68 |
| Patient 7  (S80274) | 10 ng (serum) | 4,176,953 | 355.97 | 8,671,157 | 2,918.10 |
| Patient 8  (K93878) | 101 ng (serum) | 5,313,685 | 1,776.08 | 4,073,252 | 1,220.99 |
| Patient 9  (D89802) | 45 ng (serum) | 4,967,670 | 1,764.53 | 8,297,537 | 2,816.02 |
| Patient 10  (M71182) | 10 ng (plasma) | 5,628,552 | 1,801.65 | 8,752,734 | 2,328.37 |
| Patient 11  (M85099) | 10 ng (serum) | 5,081,025 | 532.79 | 5,580,422 | 1,854.43 |
| Patient 12  (P73793) | 10 ng (plasma) | 5,121,630 | 352.90 | 7,405,013 | 2,660.17 |
| Patient 13  (B68225) | 117 ng (serum) | 5,707,795 | 882.45 | 7,562,846 | 2,455.77 |
| Patient 14  (B78899) | 17 ng (plasma) | 5,331,490 | 610.27 | 7,193,646 | 2,536.12 |
| Patient 15  (B62630) | 68 ng (serum) | 5,368,224 | 938.61 | 4,460,607 | 1,435.68 |
| Patient 16  (T73616) | 39 ng (plasma) | 3,868,785 | 1,240.69 | 3,900,340 | 1,347.22 |
| Patient 17  (S69308) | 17 ng (plasma) | 5,536,747 | 1,813.84 | 6,982,961 | 2,447.13 |
| Patient 18  (E78569) | 80 ng (plasma) | 3,391,998 | 1,115.87 | 3,872,538 | 1,281.61 |
| Patient 19  (S73507) | 30 ng (plasma) | 6,088,580 | 689.97 | 6,700,776 | 2,335.01 |

**Supplementary Table 6.**
